# Coronary physiological assessment in the catheter laboratory: haemodynamics, clinical assessment and future perspectives

**DOI:** 10.1136/heartjnl-2020-318743

**Published:** 2022-06-29

**Authors:** Paul D Morris, Rasha Kadem Al-Lamee, Colin Berry

**Affiliations:** 1 Department of Infection, Immunity and Cardiovascular Disease, The University of Sheffield, Sheffield, UK; 2 National Heart and Lung Institute, Imperial College London, London, UK; 3 BHF Glasgow Cardiovascular Research Centre, University of Glasgow, Glasgow, UK; 4 Department of Cardiology, Golden Jubilee National Hospital, Clydebank, UK

**Keywords:** Microvascular Angina, Coronary Angiography, Angina Pectoris

**Learning objectives**

Based on the *ESC Core curriculum: Section 2.9: Chronic ischaemic heart disease*

**Knowledge**

- The haemodynamic concepts and physiological principles on which invasive coronary physiological assessment is based.
- How invasive coronary physiological assessment can be used to diagnose chest pain and ischaemic heart disease.

**Skills**

- Be able to select, use and interpret invasive diagnostic tools for the evaluation of ischaemia.
- Be able to interpret the results of commonly used invasive coronary physiological assessment to optimally guide treatment including coronary revascularisation.

**Behaviours and attitudes**

- To apply an evidence-based approach to invasive physiological coronary assessment.
- To accurately interpret and communicate findings of coronary physiological assessment to the heart team.

## Introduction

Historically, invasive coronary angiography provided what was once considered the gold-standard assessment of ischaemic heart disease (IHD). The presence of epicardial coronary artery disease (CAD) was the reference standard for non-invasive tests of ischaemia. Coronary angiography alone, however, is an unreliable predictor of the ischaemia causing potential of epicardial CAD.^1^ Moreover, epicardial atherosclerotic disease is one of several causes of myocardial ischaemia.^2^ Other causes, such as those originating in the microvascular compartment and vasospastic angina, are not revealed using standard invasive or CT coronary angiography, potentially leading to inaccurate or ‘false negative’ diagnosis and suboptimal treatment.^3^ Consequently, over the last two decades, the use of intracoronary physiological assessment has increased. These tests have improved diagnostic accuracy, are associated with superior clinical outcomes and are now acknowledged in the major international guidelines.^4^ Nonetheless, more clinical evidence will be needed for cardiologists to routinely adopt coronary function testing during invasive management, notably for disease-modifying medical therapy for microvascular angina, and clinical trials are ongoing.^5 6^

More recent developments are providing fresh insight into the aetiology and pathophysiology of IHD that go well beyond the simple presence or absence of occlusive epicardial disease. This Education in Heart article summarises the fundamental haemodynamics, the enabling wire-based sensor technology, the physiological indices used in the catheterisation laboratory, the invasive assessment of ischaemia with and without obstructive coronary artery disease (INOCA) and the emerging concepts and technologies likely to impact practice in the near future.

## Coronary haemodynamics

A basic appreciation of the laws of vascular haemodynamics is useful to understand the derivation, application and interpretation of coronary physiology assessment in clinical practice. The hydraulic equivalent of Ohm’s law describes how a difference in pressure (dP) drives coronary blood flow (CBF) through the circulation, regulated by the resistance (R):

$$CBF=\frac{dP}{R}$$

Within the coronary microcirculation (CMC), the arterioles act as the ‘resistance vessels’, constantly modifying their smooth muscle tone and therefore diameter, in response to fluctuations in perfusion pressure and the concentration of metabolic products so that CBF is matched closely to the prevailing metabolic demands of the myocardium. When the arterioles are maximally dilated, coronary microvascular resistance (CMVR) is minimal and CBF is maximal (hyperaemia). At this point, CBF is related linearly to the driving pressure. The fold increase in CBF between resting (baseline) conditions and hyperaemia is the basis of coronary flow reserve (CFR):

$$CFR=\frac{CBF_{hyperaemia}}{CBF_{baseline}}$$

In unobstructed coronary arteries, there is a gradual loss of pressure due to *viscous friction* between concentric ‘laminae’ of flowing blood. Poiseuille’s law shows how pressure loss ( dP ) is related linearly to blood viscosity ( μ ), flow rate (Q) and vessel length (L).

$$dP=\frac{8\mu QL}{\pi r^{4}}$$

With flow as the subject of the equation, Poiseuille’s law explains why CBF is extremely sensitive to even small changes in arteriolar tone, being proportional to the fourth power of vessel radius (r).

$$\text{Q}=\frac{\text{dP}\Pi {\text{r}}^{4}}{8\mu \text{L}}$$

In a stenosed artery, pressure loss also occurs due to *convective acceleration* of blood. As CBF accelerates into the stenosis (to conserve total energy), there is a corresponding decrease in local pressure, as potential energy (pressure) is converted into kinetic energy. Bernoulli’s law shows how this pressure loss is proportional to blood density and to the square of the increase in velocity (V).

$$\text{dP}=\frac{1}{2}\rho \left({\text{V}}_{2}^{2}-{\text{V}}_{1}^{2}\right)$$

**Figure 1:**
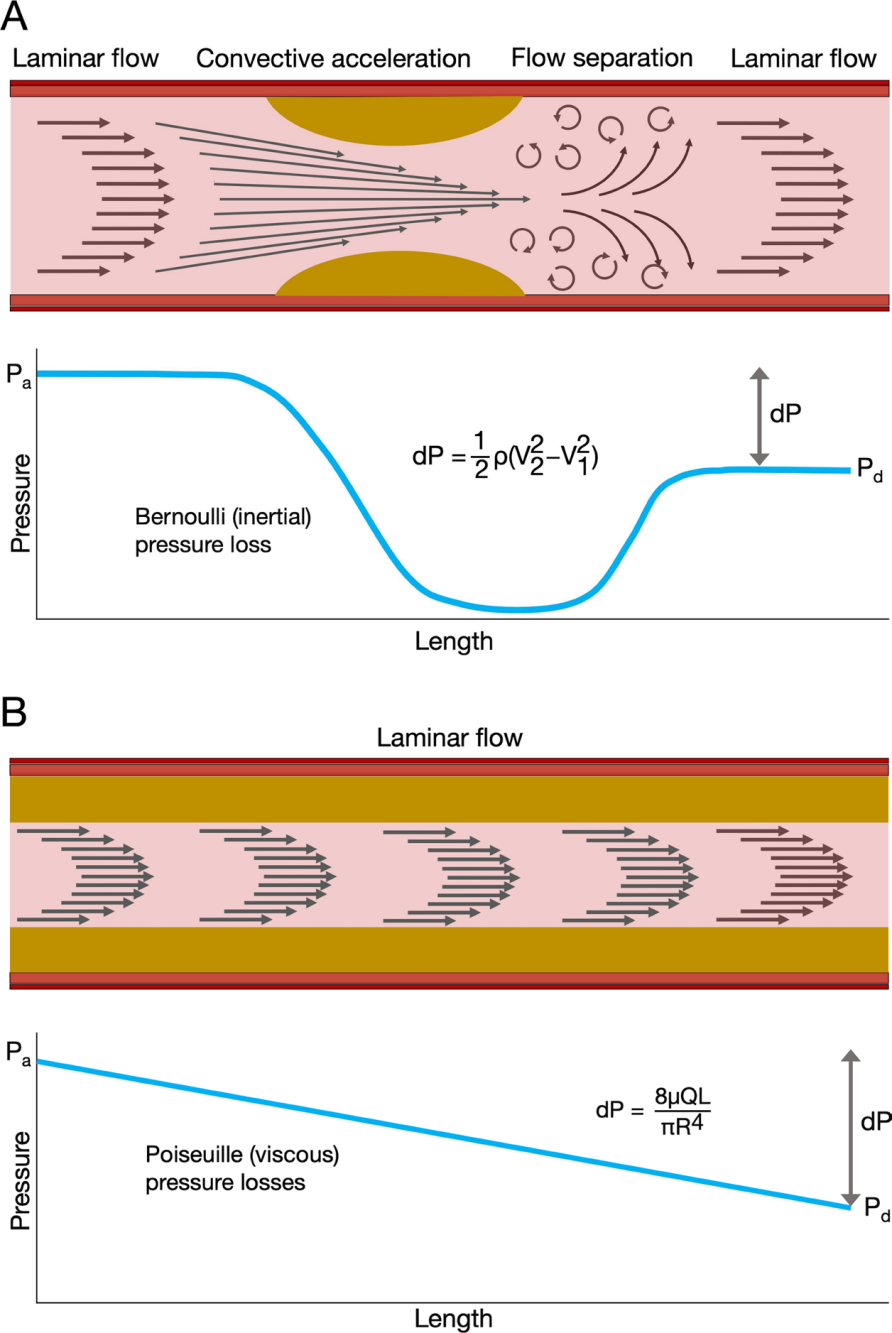
Coronary haemodynamics and pressure loss in focal (A) and diffuse (B) disease. In panel A: a focal stenosis causes acceleration of coronary blood flow (CBF) with a corresponding drop in pressure (P). In the poststenosis region, pressure recovers due to the deceleration of blood flow as the radius is restored, but this is incomplete because the transfer of kinetic energy back to potential (pressure) is energetically inefficient. This causes a net pressure drop across the lesion (dP) and is the basis for fractional flow reserve (Pd/Pa). The Bernoulli equation characterises these relationships where ρ is viscosity and V is blood velocity. If the stenosis can be eradicated by stenting, CBF is fully restored. In panel B, there is diffuse disease along the length of the artery. The radius (R) is reduced, and this causes pressure loss due to increased viscous friction between the laminae of flow blood (arrows). Viscous pressure loss is minimal in undiseased arteries but becomes significant in diffuse disease being sensitive to the fourth power of the radius. Thus, if the radius is reduced to 50%, the pressure loss is increased 16-fold over the length (L) of the disease, for a given blood density (μ) and flow rate (Q). Like focal disease, this causes pressure loss and a reduction in fractional flow reserve but is often not as amenable to stenting.

In the poststenosis region, as normal vessel calibre is restored, Bernoulli’s law explains the recovery of blood pressure, as CBF velocity reduces. Total pressure loss can be approximated as the sum of the viscous (Poiseuille) and inertial (Bernoulli) losses (figure 1).

Healthy tapering of coronary artery diameter (D) is haemodynamically distinct from a stenosis. Tapering is necessary to maintain a constant CBF velocity and wall shear stress (the frictional force exerted on the luminal surface by flowing blood), to counteract CBF lost to side branches, thus providing the lowest energy state, balancing the energy required drive blood through the coronary circulation (vascular resistance) against that required produce and maintain the blood and vessels themselves (vascular volume). Murray’s law ( ${\text{D}}_{\text{parent}}^{\text{3}}={\text{D}}_{\text{branch1}}^{\text{3}}\text{+}{\text{D}}_{\text{branch2}}^{\text{3}}$ ) and Finet’s law ( ${\text{D}}_{\text{parent}}\text{=0.678(}{\text{D}}_{\text{branch1}}\text{+}{\text{D}}_{\text{branch2}}\text{)}$ describe this counterpoise, quantify arterial taper and are helpful in relating physiology to anatomy, particularly around bifurcations.

In healthy, unobstructed arteries, CBF is governed by Poiseuille’s law, and pressure loss is minimal. In stenosed arteries, pressure loss and CBF become dominated by Bernoulli’s law. Although useful in understanding physiological assessment, these simple, analytical laws are based on assumptions not entirely satisfied in human CBF. Bernoulli’s law, for example, accurately predicts pressure drop as CBF accelerates, but would predict full pressure recovery (zero net pressure drop) distal to a stenosis when the reference diameter is restored. In reality, this does not happen, because the transfer of kinetic energy back to potential energy is inefficient due to the formation of eddy currents and flow vortices, that is, a breakdown of energetically efficient normal laminar flow. The net pressure loss is the basis of fractional flow reserve (FFR), used routinely to determine physiological lesion significance of epicardial disease from the translesional pressure gradient. According to Ohm’s law (previously), if variability in resistance can be eradicated (ie, during pharmacologically induced hyperaemia), CBF becomes proportional to the pressure gradient and so can be used as a surrogate for CBF. Thus, where Pd and Pa are the pressures distal and proximal to a stenosis:

$$\text{FFR}=\frac{{\text{CBF}}_{\text{stenosis}}}{{\text{CBF}}_{\text{no stenosis}}}\text{=}\frac{{\text{P}}_{\text{d}}}{{\text{P}}_{\text{a}}}$$

CBF predominates during diastole and is lowest during systole, the result of microvascular compression that increases CMVR. Diastolic myocardial relaxation not only reduces CMVR but generates a *backward expansion wave* that actively draws (sucks) CBF into the myocardium. This effect is more pronounced in left-sided coronaries (higher left ventricular wall pressure) and has been shown using wave intensity analysis to be significantly diminished in patients with coronary microvascular dysfunction.^7^

Ischaemia occurs when CBF is insufficient to satisfy myocardial energy demand. Most commonly, this occurs secondary to a pathological increase in resistance in either the epicardial and/or CMV compartments and may be a structural or functional, fixed or dynamic disease processes.

### Sensor-tipped wire technology

Physiological sensor technology is now incorporated into 0.014” angioplasty guidewires to measure pressure, velocity and temperature, which are substituted into relatively simple equations to calculate indices of intracoronary physiology (ICP).

Intracoronary pressure measurement is as old as PCI itself. Andreas Gruntzig used the elimination of the trans-lesional pressure gradient (Pd measured from a distal balloon port) as a marker of success in the first PCI procedure in 1977. However, it was not until piezo-electric crystal transducers were incorporated into the 0.014” guidewire in the 1990s that the concept of using pressure to guide intervention advanced into routine clinical practice. Since then, pressure-derived indices such as FFR and the non-hyperaemic pressure ratios (NHPRs) have revolutionised coronary intervention. The transducer is situated at the proximal end of the radiopaque tip (usually 30 mm from tip). Piezo-electric transducers (Abbott and Philips Volcano) provide reliable and repeatable measurements of dynamic pressure but are liable to some level of signal drift and handling characteristics are inferior to standard workhorse wires. More recently, optical fibre transducers were incorporated into 0.014” guidewires (Opsens and Boston Scientific) with some attractive handling characteristics and reduced signal drift and mounted onto microcatheters with rapid-exchange capability (Navvus II, ACIST Medical) for use over any 0.014” workhorse wire. Compared with the 0.014” wires, the increased diameter of the microcatheter causes a small but consistent (−0.02) reduction in FFR.

CBF velocity can be measured by the Doppler ultrasound using the dedicated FloWire and the ComboWire XT (both Philips). Both 0.014” wires have a 12 MHz piezo-electric pulse wave ultrasound transducer at the tip, which samples the instantaneous peak velocity (IPV) from a ‘range gate’ between 5 mm and 7 mm from the tip with a 30° arc. The ComboWire XT also incorporates a pressure transducer 15 mm from the tip for simultaneous pressure measurement. The IPV is averaged over several cardiac cycles and used to calculate the average peak velocity (APV), used as a surrogate for CBF in the calculation of CFR and hyperaemic microvascular resistance (HMR). Practice and experience are important in ensuring reliable results. The wire tip must be positioned carefully, away from the vessel wall, with coaxial alignment and fine adjustment to ensure the best signal ‘envelope’ is recorded. Signal quality can be sensitive to subtle movements of the wire, patient and the cardiac cycle itself.

When calculating CFR (ratio of hyperaemic and baseline APV), errors that are proportionally consistent between baseline and hyperaemic measurements may be balanced, but this is not the case for indices of microvascular resistance (see further), where a single value of APV is substituted. Practice and experience are important in ensuring reliable results with the Doppler wire.

Temperature-sensitive transducers are incorporated into the Abbott PressureWire X guidewire, located 30 mm from the tip, adjacent to the pressure sensor. The mean transit time (MTT) of a bolus of room-temperature saline, injected from the guiding catheter, can therefore be estimated from the temperature dynamics. The inverse of the MTT ( $\frac{\text{1}}{\text{MTT}}$ ) is used as surrogate of CBF in the calculation of CFR and the index of myocardial resistance (IMR). Saline boluses (3 mL) are injected rapidly and repeatedly, and the mean value from three consistent recordings is used. Thermodilution is generally considered less sensitive to variability in measurement technique than Doppler.

### Indices of coronary physiology

Myocardial health and function depend on CBF, and ischaemia is an insufficiency in CBF, but inconveniently, CBF cannot be measured directly. Instead, physiological assessment relies on indirect proxy markers of CBF derived from measures of pressure, flow velocity and thermodilution meant transit time.

FFR is the ratio of the pressure measured by the pressure wire distal to a lesion to that measured proximally from the guiding catheter, over the entire cardiac cycle, during hyperaemia. Hyperaemia is induced by either an intravenous infusion of adenosine at 140 µg/kg/min via a central vein (but in routine practice by a large, proximal, peripheral vein) or by an intracoronary bolus of adenosine through the guiding catheter (40 µg right and 80 µg left coronary artery). FFR is measured during maximal stable hyperaemia in the former approach and as the lowest recorded Pd/Pa ratio in the latter approach. Both approaches generate similar results, but intracoronary administration is shorter lived, associated with less adenosine-related side effects.

Using pressure as a surrogate for flow reserve requires microvascular resistance to be minimal, hence the need for hyperaemic induction. It is important to equalise the wire pressure signal to that at the catheter tip before measurement. During measurement, plugging of the guiding catheter must be avoided and the transducer should be placed ≥15 mm distal to any stenosis. After measurement, the transducer should be pulled back to the catheter tip to exclude signal drift. If the signal has drifted, the wire should be re-equalised and measurements repeated. FFR’s considerable strength is that it helps quantify CBF reduction due to coronary disease, allowing PCI to be targeted at the most flow limiting stenoses. FFR reports the fraction (percentage) of CBF, relative to a hypothetically normal artery. Thus, an FFR of 0.80 indicates a 20% reduction in flow compared with the same artery hypothetically free from disease. The original DEFER trial demonstrated the safety of deferring PCI when FFR was >0.75.^8^ The clinical threshold for determining physiological significance increased to ≤0.80 in the seminal FAME trial in which FFR-guided PCI was superior to angiography-guided PCI in reducing major adverse cardiovascular events (MACE), and in the FAME-2 trial where PCI was associated with reduced urgent revascularisation in physiologically significant lesions compared with medical therapy.^9 10^ FFR has gained a class I (A) recommendation for determining haemodynamic significance of intermediate epicardial disease and a IIa (B) recommendation for guiding PCI in multivessel cases.^11^ FFR has become established as the gold-standard invasive method for determining epicardial lesion significance, guiding PCI and as a benchmark against which other methods are assessed and validated. FFR is considered safe for use in left main stem disease, rationalises three-vessel *angiographic* disease to *physiologically* zero-vessel, one-vessel or two-vessel disease in up to 86% and can be useful in assessing bystander disease in acute coronary syndromes.^12–14^ FFR does, however, have some limitations: it is limited to epicardial coronary assessment, will be increased (more likely to be negative) in the context of reversible microvascular dysfunction, suboptimally discriminates serial lesions, cannot quantify absolute changes in CBF and exhibits small variability on repeat testing.

Non-hyperaemic pressure ratios (NHPRs), also referred to as resting indices, measure the Pd/Pa ratio during diastole when microvascular resistance is stable. Like FFR, the NHPRs are also derived from the translesional pressure gradient, but with no requirement for hyperaemic induction, they are quicker, simpler and cheaper to use compared with FFR, are not associated with adenosine-related side effects, and increase patient comfort. Although not 100% physiologically equivalent to FFR, results from landmark outcomes trials demonstrated that the original NHPR, instantaneous wave-free ratio (iFR, Philips, The Netherlands), was non-inferior to FFR when used to guide PCI.^15 16^ Multiple NHPRs are now available (table 1). Despite differences in their calculation, they are numerically equivalent^17^ and have a common threshold for significance (≤0.89). The absence of hyperaemia reduces complex, non-linear, interlesion haemodynamic interactions, thus simplifying between-lesion interpretation during pull-back assessment.^18^ Similar to FFR, NHPRs are also limited to epicardial coronary assessment and do not quantify absolute CBF changes.

**Table 1:** Comparing alternative non-hyperaemic pressure ratio (NHPR) systems

Acronym	Full name	Manufacturer	Method/notes
iFR	Instantaneous wave-free ratio	Philips Healthcare	The original NHPR. Measures Pd/Pa ratio during the wave-free period during diastole when microvascular resistance is stable and minimal. Can be coregistered to the angiogram using the Philips IntraSight platform.
RFR	Resting full-cycle ratio	Abbott	Reports the lowest value of Pd/Pa across the whole cardiac cycle (but which occurs during diastole). Measured with the PressureWire X guidewire and the wireless connection to the QUANTIEN measurement system.
DFR	Diastolic hyperaemia-free ratio	Boston Scientific	Samples the Pd/Pa ratio when pressure is below the mean aortic pressure and reducing. Averaged over five cardiac cycles. Measured with COMET II wire with Asahi (INTECC USA, INC) tip for improved handling.
dPR	Diastolic pressure ratio	ACIST Medical Systems, Inc	Pd/Pa ratio at the pressure peak-to-peak midpoint, averaged over five consecutive beats. Signal (not ECG) triggered. Measured with the Navvus rapid exchange (RXi) (over-the-wire) microcatheter system allowing use of workhorse guidewire.
dPR	Diastolic pressure ratio	Opsens Medical	Measured with second-generation fibre-optic technology (Optowire) with minimal signal drift, nitonol core and ‘workhorse’ guidewire properties.

Box 1 **International standardisation of diagnostic criteria for microvascular angina according to the Coronary Vasomotion Disorders International Study (COVADIS) Group**

**Criteria**

1. Symptoms of myocardial ischaemia with:
  - Effort and/or rest angina.
  - Angina equivalents (eg, dyspnoea).
2. Absence of obstructive epicardial disease (<50% diameter stenosis or fractional flow reserve >0.80) with:
  - Coronary CT coronary angiography (CTCA).
  - Invasive coronary angiography.
3. Objective evidence of myocardial ischaemia with:
  - Ischaemic ECG changes during an episode of chest pain.
  - Stress-induced chest pain and/or ischaemic ECG changes in the presence or absence of transient/reversible abnormal myocardial perfusion and/or wall motion abnormality.
4. Evidence of impaired coronary microvascular function with one or more of:
  - Impaired CFR.
  - Coronary microvascular spasm, defined as reproduction of symptoms, ischaemic ECG changes, but no epicardial spasm during acetylcholine (ACh) testing.
  - Abnormal coronary microvascular resistance indices (eg, index of microvascular resistance >25 or hyperaemic microvascular resistance >2.5).
  - Coronary slow flow, defined as thrombolysis in myocardial infarction frame count >25.

**Diagnosis of microvascular angina**

*Definitive* if all four criteria are met.

*Suspected* if criterion 1+2 are met, but only three or four are also met.

Adapted from Ong *et al.* 29

CFR is the ratio of hyperaemic to baseline CBF and is distinct from FFR and NHPRs in two important ways. First, CFR assesses the combined effects of the epicardial and microvascular physiology but cannot differentiate between the two. Second, CFR reports the fold increase in CBF from resting conditions to hyperaemia and therefore, also reflects the vasodilatory reserve of the microvasculature. Doppler velocity or thermodilution-derived MTT are used as surrogates for CBF. The former is therefore, more accurately referred to as coronary velocity flow reserve (CFVR).

$$CFVR=\frac{APV_{hyperaemia}}{APV_{baseline}}$$

$$CFR_{thermodilution}=\frac{MTT_{baseline}}{MTT_{hyperaemia}}$$

An abnormal CFR is considered <2.5 (<2.0 by some thermodilution protocols), but in healthy individuals, may exceed 4.0, and in some reach 6.0.^4^ A recent metanalysis of 79 studies and nearly 60 000 patient cases demonstrated that each 0.10 reduction in CFR, below normal, was associated with a 16% increase in mortality and 8% increase in MACE.^19^ CFR is particularly useful when combined with pressure measurement to help discriminate the contribution of epicardial and microvascular disease.^20^ CFR may be reduced erroneously in patients with increased baseline cardiovascular workload or anxiety who are not truly at their cardiac baseline. This effect may be reduced by coronary flow capacity (CFC), which integrates CFR with the hyperaemic APV to create a two-dimensional CFC ‘map’, which may increase diagnostic and prognostic power beyond CFR alone.^21^

IMR and hyperaemic microvascular resistance (HMR) are both markers of the minimal CMVR. Ohm’s law ( $\text{R=}\frac{\text{dP}}{\text{CBF}}$ ) demonstrates how simultaneous measurement of pressure gradient and CBF allows resistance to be calculated ( $\text{CMVR=}\frac{\text{dP}}{\text{CBF}}$ ). For CMVR, the pressure gradient is taken as the distal epicardial pressure because right atrial pressure is assumed to be negligible. Doppler-derived APV or (the inverse of) thermodilution-derived MTT are substituted to represent CBF. If the former is used, it is known as HMR and if the latter, IMR.

$$\text{IMR=}{\text{P}}_{\text{d}}\text{∙MTT}$$

$$IMR=p_{d}\cdot MTT$$

**Figure 2:**
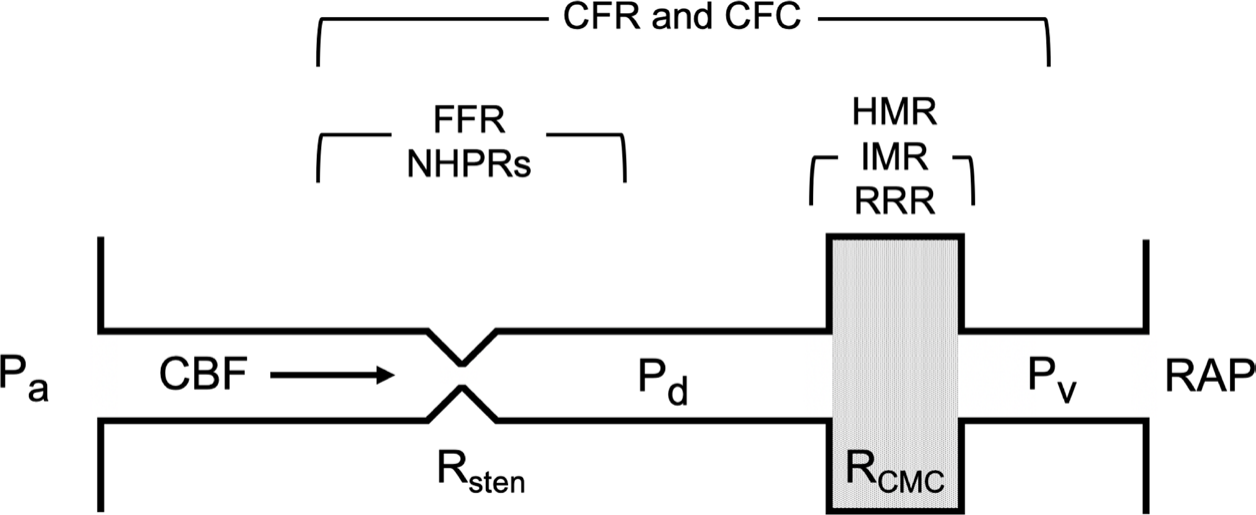
Schematic summarising the commonly used coronary physiological indices. CBF, coronary blood flow; CFC, coronary flow capacity; CFR, coronary flow reserve; FFR, fractional flow reserve; HMR, hyperaemic microvascular resistance; IMR, index of microvascular resistance; NHPRs, non-hyperaemic pressure ratios; Pa, proximal (aortic) pressure; Pd, distal coronary arterial pressure; PV, coronary venous pressure; RAP, right atrial pressure; R_CMC_, resistance of the coronary microcirculation; RRR, relative resistance ratio; Rsten, stenosis resistance.

**Figure 3:**
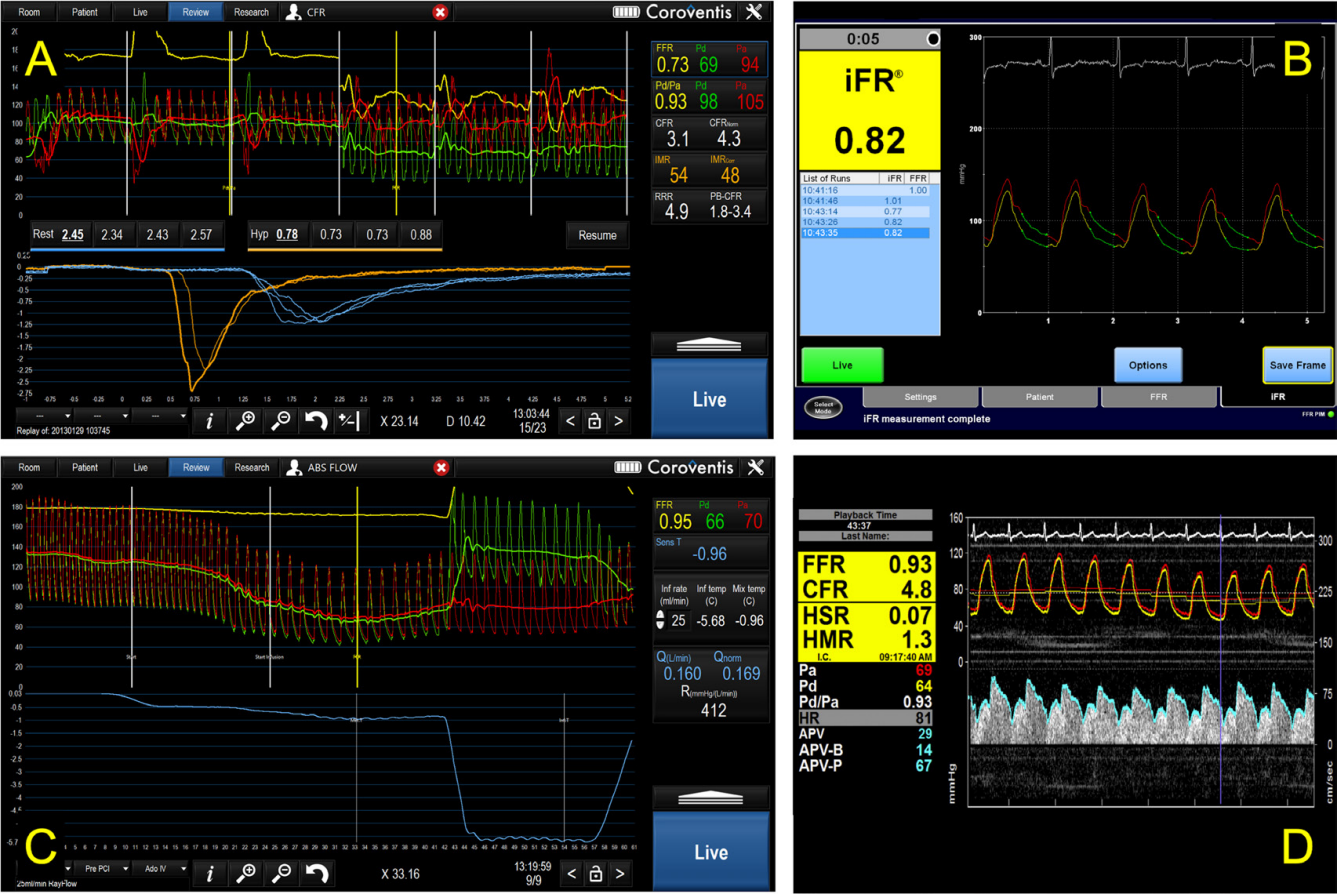
Examples of invasive coronary physiological assessment in the cardiac catheter laboratory. Panel A demonstrates fractional flow reserve (FFR), resting Pd/Pa ratio, coronary flow reserve (CFR), index of microcirculatory resistance (IMR) and the corresponding thermodilution curves (lower half panel A). The proximal and distal pressure signals (red and green) are seen in the upper half the panel. The relative resistance ratio is also displayed. Panel B demonstrates instantaneous wave-free ratio, a non-hyperaemic pressure ratio. Panel C demonstrates investigation of absolute coronary blood flow (L/min) and absolute microvascular resistance (mmHg/(L/min)). Panel D demonstrates FFR and Doppler-derived CFR, HSR and HMR. Panel D is reproduced (unedited) from Ahmed *et al*, Elsevier (https://doi.org/10.1016/j.jcin.2018.07.019) under a Creative Commons 4.0 licence. HMR, hyperaemic microvascular resistance; HSR, hyperaemic stenosis resistance; Pa, proximal (aortic) pressure; Pd, distal coronary arterial pressure.

IMR is normally <25 and HMR <2.5.^3 4 22^ These indices estimate the resistance to CBF only in the microvascular compartment. This is distinct to CFR which, even when abnormal, cannot localise if the pathology is in the epicardial and/or the microvascular compartment. The ratio of hyperaemic and baseline IMR is known as the resistive reserve ratio (RRR) which, like CFR, reflects vasodilatory capacity. Translesional (epicardial) resistance can also be quantified by applying Ohm’s law, as the ratio of Pa-Pd and CBF, referred to as hyperaemic or baseline stenosis resistance (HSR and BSR) depending when measured. Neither are used routinely but are interesting areas for research and development. Figures 2 and 3 demonstrate the commonly used physiological indices schematically and during catheter laboratory measurement.

### Absolute physiology

**Figure 4:**
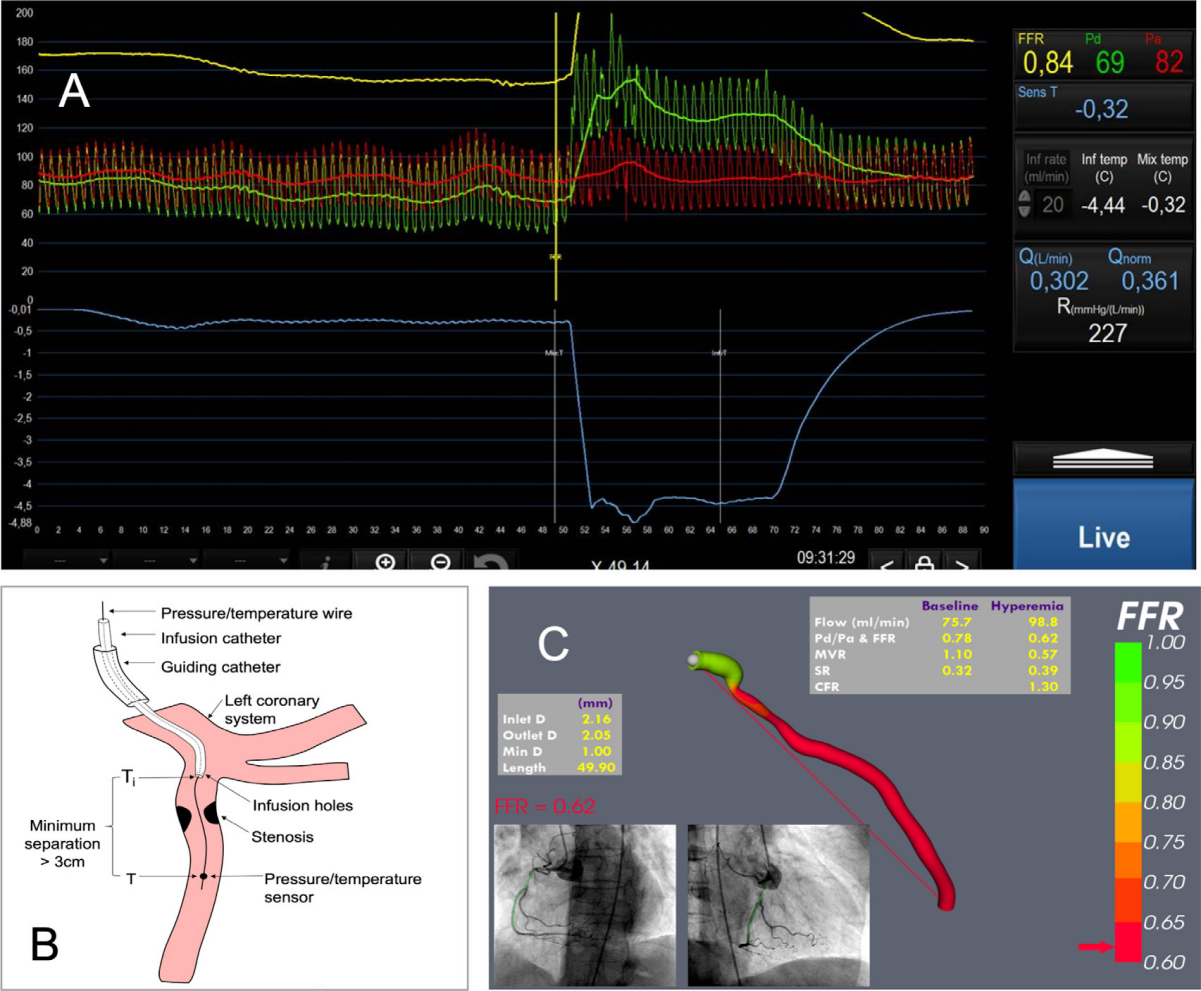
Absolute coronary physiology. Panel A demonstrates a measurement of absolute coronary blood flow (Q), the flow normalised for the presence of the Rayflow catheter (Qnorm) and the distal resistance (R) using the continuous infusion thermodilution method. From left to right: as room temperature saline is infused from the proximal Rayflow catheter, hyperaemia is induced (after ~20 s), the proximal and distal pressure signals (red and green) begin to separate and the distal temperature gradually falls (light blue line). The sudden drop in temperature (middle) is where the transducer is retracted to the catheter tip to measure the infusion temperature. Once this is measured, the infusion is stopped, and the temperature returns to baseline. The final measurements are displayed in the right-hand panel. The flow that is estimated is equal to the flow distal to the tip of the infusion catheter. Panel B demonstrates schematically the arrangement of the distal pressure wire location and the more proximal Rayflow infusion catheter. Ti indicates the infusion temperature of saline, and T indicates the fully mixed temperature. Panel C demonstrates the virtuQ system (University of Sheffield) for deriving absolute coronary flow, microvascular resistance (MVR), stenosis resistance (SR) and coronary flow reserve (CFR) from a computational fluid dynamics simulation based on the reconstructed arterial anatomy. Panel A courtesy of Dr Daniëlle Keulards, Catherina Hospital, Eindhoven, The Netherlands. Panel B courtesy of Dr Daniel Taylor, University of Sheffield, UK. Panel C courtesy of Dr Louise Aubiniere-Robb, University of Sheffield. FFR, fractional flow reserve.

It is also possible to predict CBF and CMVR in absolute units (mL/min and mmHg.min/mL, respectively). The continuous infusion thermodilution method uses a dedicated, rapid-exchange Rayflow catheter (Hexacath, Paris, France) to infuse room temperature saline from the proximal portion of the artery being investigated. Temperature change is detected by the wire-mounted transducer placed ≥3 cm distally, the magnitude of which allows the prediction of artery-specific absolute CBF (aCBF) and absolute CMVR (aCMVR).^23 24^ The virtuQ method computes aCBF, aCMVR and CFR by applying a numerical solution to a three-dimensional (3D) arterial reconstruction. It does not require dedicated hardware aside from a standard pressure wire but is an academic application and not available commercially.^25^ After years of relying on indirect surrogate markers, the ability to determine aCBF and aCMVR does appear attractive, especially when complemented with pressure measurements because this enables a comprehensive assessment of epicardial and microvascular physiology in a single test in absolute units (figure 4).

Threshold values for physiological indices are based on observational and trial data, as the point that, when crossed, is most predictive of ischaemia or excess MACE. Those quoted in this manuscript are widely accepted and consistent with the ESC guidelines.^4^ Single ‘cut-off’ values are helpful in guiding decision making but are a single point along a physiological continuum, balance sensitivity against specificity and should not replace good clinical judgement.

### Assessing the CMC and vasoreactivity

The coronary microvasculature cannot be seen, stented or instrumented in the catheter laboratory and, in terms of perceived clinical importance and research effort, has lagged well behind epicardial CAD. The microvasculature, however, regulates overall coronary physiology and is the location for pathological changes causing ischaemia with no obstructive coronary artery disease (INOCA).

INOCA is common. Almost half of all chest pain patients have no obstructive disease at angiography and of these 68% have evidence of microvascular dysfunction.^26^ Moreover, when formally assessed in the catheter laboratory, and managed with stratified medical therapy, these patients experience a reduction in symptoms and improved quality of life.^3^ INOCA patients are three times more likely to be female and are at increased risk of major adverse cardiovascular events. Yet, following standard angiography with or without FFR assessment, these patients are often reassured and discharged with no diagnosis or treatment.^27^ This leads to a reduction in quality of life and mental health, persistence of symptoms, representation to cardiac services (~50%) and increased healthcare costs.^28^ INOCA may occur as a primary CMC problem or secondary to conditions that cause ventricular hypertrophy, infiltration or inflammation. It may be a structural or functional CMC disorder.

**Figure 5:**
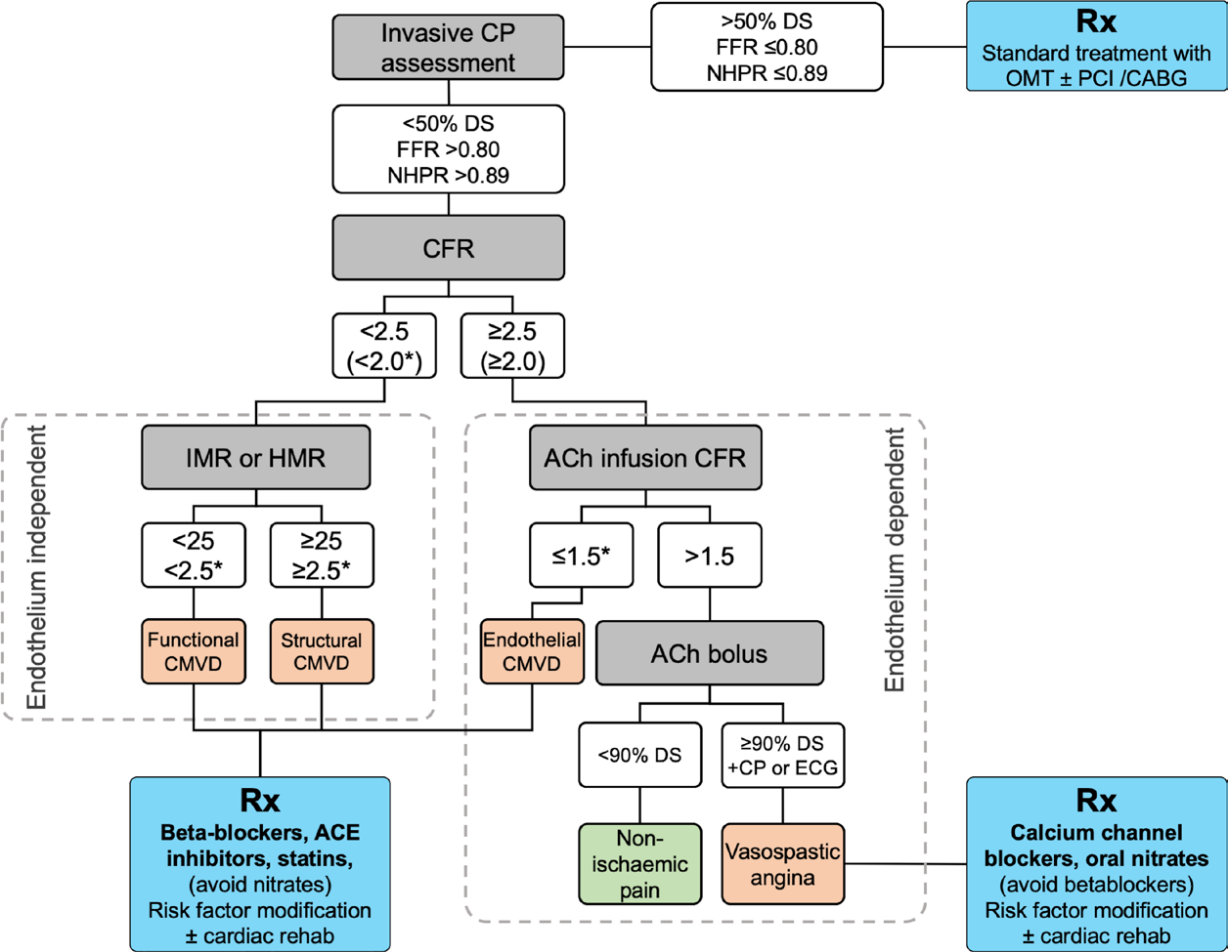
An approach to intracoronary physiological assessment for patients with chest pain but no obstructive coronary disease. *Indicates range for Doppler-based CFR (used in some protocols) and IMR. The European Society of Cardiology (ESC) guidelines give a IIa (B) recommendation for measuring CFR and microvascular resistance in patients with chest pain but unobstructed arteries.^4^ ACh, acetylcholine; CFR, coronary flow reserve; CMVD, coronary microvascular dysfunction; CP, chest pain; DS, diameter stenosis; FFR, fractional flow reserve; HMR, hyperaemic microvascular resistance; IMR, index of microvascular resistance; NHPR, non-hyperaemic pressure ratio.

Combined assessment of pressure CBF is helpful to calculate CMVR (IMR or HMR) and support the diagnosis of INOCA. More advanced assessment with endothelial testing may help to elucidate the underlying mechanism. Figure 5 outlines an approach to physiological assessment of the microvasculature. Table 1 summarises the diagnostic criteria for microvascular angina in patients with evidence of INOCA.^29^ Epicardial and microvascular disease are not mutually exclusive and frequently coexist. This may help to explain the roughly 20% of patients who do not gain full symptomatic relief after FFR-guided PCI.^30^ Using the thermodilution method (Abbott pressure wire X), CFR, IMR and RRR can all be measured with no additional hardware and minimal extra on table time.

### Intracoronary ACh

Whereas adenosine tests endothelium-independent vasodilation, ACh acts on the endothelium to release nitric oxide causing vasodilation. Intracoronary ACh is therefore, used to identify endothelial dysfunction. Intracoronary ACh testing is used in specialist centres and is currently an off-label indication. Under low dose ACh infusion, failure of the CFR to rise above 1.5 supports a diagnosis of endothelial dysfunction, a known cause of microvascular angina (figure 5). At higher doses, ACh also acts directly on vascular smooth muscle cells to cause vasoconstriction, and this is used to provoke epicardial spasm in patients suspected to have vasospastic angina. This is associated with high diagnostic accuracy (sensitivity 90%, specificity 99%).^4 31^ Vasospastic angina causes ischaemic chest pain, typically at rest, without effort intolerance. Patients tend to be younger and, aside from smoking, may lack classical cardiovascular risk factors. Diagnostic criteria are summarised in box 2.^32^

Box 2 **The international standardisation of diagnostic criteria for vasospastic angina according to the Coronary Vasomotion Disorders International Study (COVADIS) Group**

**Criteria**

1. Nitrate-responsive angina, during spontaneous episode, with one or more of:
  - Rest angina, especially between night and early morning.
  - Marked diurnal variation in exercise tolerance—reduced in morning.
  - Hyperventilation can precipitate an episode.
  - Calcium channel blockers (but not β-blockers) suppress episodes.
2. Transient ischaemic ECG changes during spontaneous episode, including any of the following in two or more contiguous leads:
  - ST segment elevation ≥1 mm (≥0.1 mV).
  - ST segment depression ≥1 mm (≥0.1 mV).
  - New negative U waves.
3. Coronary artery spasm: defined as transient total or subtotal coronary artery occlusion (>90% constriction) with angina and ischaemic ECG changes either spontaneously or in response to a provocative stimulus (typically ACh, ergonovine/ergometrine or hyperventilation).

**Diagnosis of vasospastic angina**

*Definitive* if criterion 1 is met + either 2 *or* 3 are also met.

*Suspected* if criterion 1 is met but 2 and 3 are equivocal.

Adapted from Beltrame *et al.* 32

The ESC guidelines advocate that guidewire-based measurement of CFR and/or microcirculatory resistance should be considered (class IIa) in those with chest pain but no obstructive epicardial disease, and that ACh testing should be considered (class IIa) in patients with suspected vasospastic angina.^4^ These more advanced assessments help to distinguish true non-cardiac pain from coronary microvascular dysfunction and vasospastic angina, ensuring more precision in diagnosis and targeted evidence-based therapy.

### Wireless intracoronary physiology

**Figure 6:**
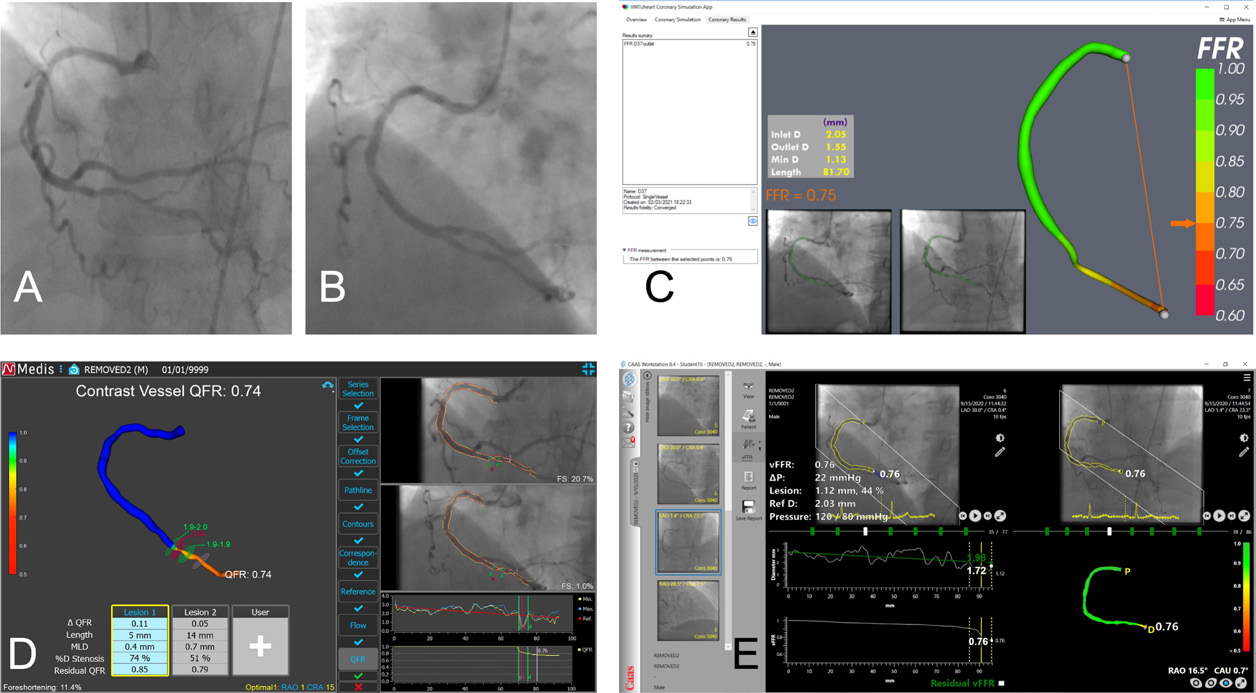
Angiography-derived (virtual) fractional flow reserve (vFFR). A diseased posterior descending artery has been processed using three different wireless FFR systems. Two angiogram images (A and B) have been used to reconstruct the three-dimensional coronary anatomy in each case. The VIRTUheart system (C; University of Sheffield, UK) has calculated vFFR at 0.75. The Medis Medical Imaging Systems (BV, The Netherlands) has calculated quantitative flow ratio (QFR; analogous to vFFR) as 0.74 (D). The CAAS workstation has calculated vFFR at 0.76 (E). Each system displays the angiogram images used, the reconstructed artery and the local diameter data within a user-friendly and interactive graphical environment.

Angiography-derived physiological assessment involves the operator reconstructing the 3D coronary anatomy from two angiographic projections ≥30° apart. A physics-based, mathematical solution is then applied to calculate the translesional pressure drop and the ‘virtual’ FFR (vFFR). Several academic and commercial methods are available. vFFR results have 95% limits of agreement of around FFR ±0.14 (figure 6).^33^ Without the associated additional time, effort and cost of deploying a pressure wire, this approach is clearly attractive and may extend physiological assessment to many more patients. However, the lack of invasive data means that assumptions have to be made in the calculation, particularly regarding the CMVR, an important determinant of FFR, and the reason that physiological assessment is different to the anatomy seen at angiography. Failure to fully account for this may reduce the physiological value of vFFR. These methods require experience and training to ensure accuracy and to minimise interobserver variability.^34^ The FAVOR III China study was the first trial to demonstrate improved clinical outcomes associated with vFFR (compared with angiography guidance).^35^ Several more clinical trials of different vFFR systems are expected to report over the next 2 years, mostly focused on demonstrating non-inferiority against invasive FFR.

### Future perspectives

Although useful in guiding PCI in intermediate cases, pressure-derived FFR and NHPRs are limited to epicardial physiology and apply a single threshold for significance, for all arteries, in all patients, under all circumstances. This is likely to evolve such that physiological evaluation will become personalised with physiology interpreted as a continuous metric that is more disease, patient and presentation specific. A move towards combined assessment of pressure and CBF would facilitate this by incorporating microvascular physiology. The ability to diagnose and phenotype patients with INOCA is stimulating more clinical trials, such as the International Coronary Microvascular Angina (iCorMicA trial) (NCT04674449) and therapy development using novel compounds with disease-modifying potential, such as an oral endothelin A receptor selective antagonist (NCT04097314) and novel devices, such as the coronary sinus reducer.^5^ In the UK, the Coronary Microvascular Dysfunction workstream of the National Institute for Health and Care Research and British Heart Foundation (NIHR-BHF) Partnership has outlined standard operating procedures to facilitate accurate disease characterisation, tailored therapies and consistent data collection for collaborative research.^36^ Learning from the success of FFR, future innovations need to be evidence based, reproducible and relatively simple to measure, interpret and apply. Non-invasive testing, performed prior to the catheterisation laboratory, may help to gate-keep, stratify and tailor an individualised assessment approach.^37^ More advanced, second-line testing may initially be performed in tertiary centres by interventionists with specialist training. However, the equipment, methods and interpretation are only a small advance from more routine testing of indices such as FFR and so these methods may soon be performed more widely. Computer and simulation-based solutions are exciting and have lots of potential, but physiology cannot be computed solely from anatomy and so it is vital these systems incorporate sufficient patient-specific information to provide genuine physiological value. Future innovations will coregister physiological assessment with imaging techniques like angiography and intravascular imaging, within intuitive systems allowing anatomy and physiology to be assessed simultaneously. Longer term, it will be interesting to see if and how biological markers of risk and prognosis can be similarly incorporated into a truly comprehensive assessment.

## Conclusions

Invasive physiological assessment has become established as an important component of patient assessment in the cardiac catheterisation laboratory in selected patients. Based on fundamental haemodynamic principles, a large and growing number of physiological indices are available. These methods are improving our understanding of IHD, and how it is best treated, on an individual patient basis. Physiological assessment is advancing rapidly, allowing a more comprehensive and detailed assessment that incorporates the CMC and endothelial function testing, which is particularly helpful when assessing patients with INOCA.

**Key points**

- Coronary physiological assessment plays an important evidence-based role in the assessment of patients in the cardiac catheter laboratory.
- Assessments are based on fundamental haemodynamic principles that are helpful in understanding the rationale, derivation and interpretation of physiological indices.
- Each assessment tests a different component of coronary physiology, and each has individual strengths and weaknesses.
- Assessment of coronary blood flow is usually derived from surrogate markers of flow, but newer methods are enabling absolute flow and resistance to be measured.
- Combined assessment of pressure and flow facilitates a more comprehensive assessment of coronary physiology and may be particularly useful in diagnosing ischaemia with no obstructive coronary disease.
- Wireless techniques derived from anatomy are attractive because they are less invasive but they must incorporate patient-specific physiology.

**CME credits for Education in Heart**

Education in Heart articles are accredited for CME by various providers. To answer the accompanying multiple choice questions (MCQs) and obtain your credits, click on the ‘Take the Test’ link on the online version of the article. The MCQs are hosted on BMJ Learning. All users must complete a one-time registration on BMJ Learning and subsequently log in on every visit using their username and password to access modules and their CME record. Accreditation is only valid for 2 years from the date of publication. Printable CME certificates are available to users that achieve the minimum pass mark.
